# The State of Lunacy in England

**Published:** 1861-10

**Authors:** 


### Art. IX.—THE STATE OF LUNACY IN ENGLAND.

The Fifteenth Report of the Commissioners of Lunacy, now before us, is of unusual interest. In addition to the subjects ordinarily treated, the Report contains a series of statistical tables in continuation of those published in the Eighth Report of the Commissioners, and a discussion of the causes which give rise to a progressive increase of patients in our asylums.

It would seem, indeed, as if the Commissioners had become fully alive to the necessity of removing that unpleasant vagueness which has so long vitiated the statistics of insanity in this country, and which became so painfully, and, we may add, seriously manifest during the late Parliamentary Inquiry on Lunatics, as we had occasion to point out more than once at the time.* For example, the official evidence on the vexed question of the increase of lunacy in the kingdom left the Select Committee almost entirely in the dark, notwithstanding that the solution, or approximate solution of that question had a highly important bearing upon its deliberations. The ordinary asylum returns showed a steady increase from year to year in the amount of known lunacy. Asylum accommodation barely kept pace with this increase, leaving out of consideration altogether the large number of the insane most unfitly confined in workhouses. Was the known increase of lunacy dependent upon an actual augmentation of the number of lunatics among the population, or was it to be accounted for on other grounds? It is manifest that upon the answer to this question must, in a great measure, depend any satisfactory solution of that difficult problem, the further provision for the insane. But although it was suggested in evidence that the increased attention given to lunacy, and consequent bringing to light of cases previously unnoticed, and the possible prolongation of life among the inmates of asylums, might give rise to an accumulation of lunatics, even to an extent rendering unnecessary any supposition that there was a true and progressive increase of insanity among us, no data were produced by which the actual value of these suggestions could be tested. So far from any light being thrown upon this question by the Parliamentary Inquiry, it was if anything made more obscure.

In their present Report the Commissioners of Lunacy for the first time give us some precise information upon the probable

* See *Journal of Psychological Medicine*, vol. xii. p. 337, 387; vol. xiii. p. 437.

sources of the progressive increase in the amount of known lunacy in the kingdom. Why this information should not have been forthcoming before the Select Committee of last Session, (1860) terminated its sittings, it is difficult to surmise. Notwithstanding, however, that it is much to be regretted that the results now made known to us were not then given in evidence, we are only too glad to get hold of them to indulge in much retrospective grumbling.

In what follows on the increase of known lunacy, we shall adhere almost verbally to the Report of the Commissioners.* It may be well, however, in the first place, to indicate the importance of the subsequent details, by stating that they tend to the following conclusions:—

1. That the increase of known lunacy for several years back has been confined almost solely to pauper lunatics.

2. That the amount of known lunacy among those classes of the population who are raised above pauperism, or are liable to pauperism from lunacy,—the wealthier classes, in short,—has been diminishing, or at least has been stationary, for some years.

3. That it is probable that lunacy is not increasing in the kingdom in greater proportion than the increase of population:—the apparent increase of lunacy among us, as shown by the steadily growing population of our asylums, and the need for still more and more asylum accommodation, being dependent mainly upon certain circumstances incidental to the provision made for the care of lunatics in late years.

From the 1st of January, 1849, to the 1st January, 1859, the number of lunatics in the various asylums of England and Wales advanced from 14,560 to 22,853.

| | Jan. 1, 1849. | Jan. 1, 1859. |
|---|---|---|
| County and Borough Asylums | 6494 | 15,845 |
| Lunatic Hospitals | 1135 | 1992 |
| Licensed Houses | 6931 | 5016 |

The increase, as these figures show, was chiefly confined to public asylums, the lunatic population of the county and borough asylums having augmented 9351 in the ten years; that of the lunatic hospitals, 857; while there had been an actual decrease in the number of patients in licensed houses to the extent of 1915.

The great increase in the total number of lunatics in asylums during the ten years was limited almost entirely to pauper and criminal patients.

Within the same decennial period the number of *pauper*

* See Report, pp. 75-84.

*lunatics* detained in public and private asylums and hospitals had advanced from 10,801 to 18,022—an increase of 7221 patients. The number of pauper lunatics detained in county asylums was more than doubled during the period, the augmentation being from 6269 to 15,618; but in the same period the number of pauper lunatics in licensed houses fell from 4178 to 2188—a diminution of 1990.

The returns of *private patients* show a total increase of 1072 in the ten years; namely, from 3759 to 4831. In county asylums the number of private patients remained nearly stationary, there being an increase of only *two* cases. In licensed houses the number had advanced from 2753 to 2828; in hospitals from 781 to 1776. "But as regards the number of private patients "in licensed houses, it is necessary to bear in mind that, in the "return made in 1849, the patients in the licensed house at "Abington Abbey were then placed to the account of hospitals; "that a large number of State patients at Fisherton are now re- "garded as private patients; and that the insane belonging to the "army and navy are now also enumerated as private patients, "but were not included at all in the computation in 1849." In January, 1859, the military and criminal patients, with the inmates of Abington Abbey, amounted to more than 300, and the apparent increase of private patients in licensed houses during the ten years was only 75; hence the Commissioners arrive at the conclusion that from the year 1849 to 1859 there was a decrease of at least 225 private patients in the licensed houses of England and Wales.

As respects the apparent increase of 995 private patients in hospitals, it would appear that there has to be taken into consideration that the number includes the inmates of Bethlem Hospital, of the Idiot Institution at Earlswood, and of the Naval Hospital at Haslar, who did not appear in the return for 1849. Now, on the 1st of January, 1859, the inmates of these three institutions amounted to 788; and after deducting this number from the aggregate return of all private patients in Hospitals, it is found that the number of insane private patients in the hospitals in England and Wales had increased to the extent of about 207 cases in ten years.*

"As, therefore," say the Commissioners, "there has been only "an addition of two such cases in county asylums, and as there "has been a diminution of numbers in licensed houses to the ex- "tent of 225, we may draw the inference that, taking an aggre- "gate of the inmates of all the various asylums in England and

* In a foot-note the Commissioners say: "The number of patients in Abington Abbey being nearly equal to the military officers at Coton Hill, the subtraction of the one and the addition of the other would not affect the calculation."

"Wales, there has not been any increase in the numbers of re-"gistered private patients during the period of ten years ending "1st January, 1859.

"Thus it appears, that while in the period above referred to "pauper patients in asylums have advanced to the extent of 7221, "there has been no addition to the number of private patients."

The returns for the ten years also show a curious and note-worthy disproportion between the two sexes among the private and pauper classes of patients. As respects the former, in 1849 there were 5 more women than men, whereas in 1859 the men exceeded the women by as many as 419. This circumstance is, however, to be explained by the consideration, that at the latter period the military and naval, as well as a proportion of the State patients, were added to the number of private patients. But as respects paupers, in 1849 the women exceeded the men in number by 851, and in 1859 this disproportion amounted to no less than 1680, a result caused by the rate of mortality being lower among women than men.

We learn, therefore, from the foregoing calculations, that *while the number of pauper patients has increased so enormously, there has been no augmentation of the private class.* Nay more, the Commissioners say—

"It is to be remarked as respects the class of private patients, in reference to the circumstance that their number in licensed houses has fallen considerably during ten years, and that in asylums generally there has been no increase—that many old-standing cases not before reported are included in the returns as now made; and it is evident, therefore, that a just comparison of the relative numbers at the beginning and end of the period under review, can only be made by deducting from the present number the cases formerly omitted. By doing this, we arrive at the conclusion that the proportion of private patients in the various asylums in England and Wales has diminished during a period of ten years; and this, notwithstanding the improvements effected in those houses during that time, rendering them more comfortable and cheerful, and increasing the inducements to send patients into them.

"To ascertain, if possible, the cause of this large increase of Pauper Patients in so short a time is," say the Commissioners, "obviously important, and the questions naturally arise,—May it be attributable to an increased amount of pauperism in the country, or to a greater liability to attacks of insanity among the poorer classes, or to both these agencies combined?

"We have positive information that pauperism has decreased,*

* "The decrease in the average number of paupers of all classes in receipt of relief at one time in 1859, as compared with 1849, is 20.5 per cent.; and as regards able-bodied paupers, the decrease in 1859, as compared with 1849, is 40.7." —*Twelfth Report of the Poor-Law Board.*

and we are unable to discover any material changes in the social condition of the labouring population rendering them more prone to mental disease."

Failing, therefore, to find a satisfactory solution on the ground of increase either in pauperism or insanity among the poor, the Commissioners are driven to search for some other source of a result, which they justly characterize as so remarkable. With this view, they have taken into consideration "every circumstance "bearing directly or indirectly on the condition of the insane "poor, which may have had the apparent effect of increasing "their number," and they have classified the agencies at work under the following heads :—

1. The large number of cases previously unreported, and only recently brought under observation.

2. The increased number of those sent to asylums.

3. The prolongation of their life when thus brought under care.

Upon the first head the Commissioners say :—

"There can be very little doubt that the system of observation and inquiry adopted of late years, however imperfect it still may be, has led to the detection and classification as insane of many persons formerly looked upon as ordinary paupers. The definition acted upon by us, that all persons receiving relief on account of mental infirmity should be included in the return; our own visitation of workhouses; the payment made to district medical officers for their attendance on single patients, and for drawing out lists of them four times a year, have all been instrumental in placing on record many cases previously overlooked. Again, the annual returns required from the parochial authorities, and which form the basis of the general summary, although still incomplete, are made in a more satisfactory manner; the omissions both of individual cases from the list, and of unions making no return whatever, being less numerous now than formerly. By these means, therefore, a considerable number of patients, before passed by, have been added to the general reckoning of insane paupers.

"The erection and opening of county lunatic asylums throughout the country may also have contributed to the same end, by exciting the attention of the public to the subject, and thus tending to the discovery and presentation of fit objects for such institutions."

Upon the second and third heads the Commissioners observe :—

"We now come to consider the effect produced by the removal of patients into county asylums, and especially of those affected with a chronic form of the malady. A very large amount of additional accommodation having been thrown open during the period under review, the parochial authorities have availed themselves of it, and removed to asylums numbers of paupers who would otherwise have remained in the workhouses or cottages. Our own visitation of workhouses, and the careful examination of the Quarterly Lists of single patients, have caused the removal of many such. The result has been that a large number of pauper lunatics, previously most unfavourably circumstanced, have been placed in houses specially adapted to their cases, and calculated to promote longevity. To what actual extent this has tended to prolong life is a question not easily solved, because we are unable to discover the rate of mortality that prevailed among this class of patients before their reception into the asylums. But we are warranted in assuming that when destitute and diseased persons are placed under care in establishments well constructed, well regulated, and specially adapted for their protection and treatment, and in which they receive succour, abundant food, and careful medical supervision, the result will be a prolongation of lives which otherwise would have been of short duration. In every other situation, whether lodged in cottages or in workhouses, insane paupers are exposed to many causes of disease and death, from which in county asylums they are entirely protected.

"While estimating these effects, it is right also to bear in mind that an improvement to a certain extent in the condition of those remaining at home or in lodgings, may have been effected by the regular medical visitation of them which was instituted in 1853. In all probability, some of the causes of disease to which they were formerly exposed have been averted, and an increase of numbers arising from a less rapid removal by death may have taken place. A like result may also have arisen as respects the inmates of workhouses formerly in unhealthy and densely populated localities, but now removed to more open and salubrious sites."

In corroboration of this assumption the Commissioners allude to the relative number of insane paupers in districts provided with asylums, as compared with localities where no such provision has been made. They say :—

"If we bring into comparison the two counties of Monmouth and Hereford, having asylum accommodation, with the three counties of Cardigan, Carmarthen, and Pembroke, where no such accommodation exists, we find that the population of the two former counties is nearly equal to that of the three latter; but that where provision has been made, the number of pauper lunatics appears to be in excess in the proportion of 514 to 456; and further, that an excess of 2000 paupers in receipt of relief are reported in the district having no asylum, over the one provided with such accommodation. Thus, in two contiguous districts in South Wales, nearly equal in population, the one which has a large excess of paupers receiving relief, has at the same time a considerably smaller number of insane paupers under care.

"A similar instance may be adduced in the county of Kent. The boroughs of Maidstone and Canterbury are nearly equally pupulous. The former has during a very long period provided for its insane paupers in the neighbouring county asylum, whilst the latter has made no provision whatever, and the returns now show double the number of insane paupers in the provided over the unprovided borough."

The Commissioners then proceed;—

"Having taken into account the probable effect produced by opening county asylums, and thus bringing insane paupers under their shelter, we now enter on a branch of the subject more capable of demonstration, namely, that the diminished rate of mortality in many of our county asylums, and especially in the large pauper houses, produced in all probability by improved accommodation, food, and medical treatment received therein by the patients, has been an obvious cause of the increase in their numbers. For instance, the average annual mortality in the three metropolitan licensed houses receiving paupers, was, during the five years ending 1843, upwards of 18 per cent. of the patients resident; whereas for several years past the proportion of deaths has been so considerably reduced below this high rate as to have caused a large addition to the aggregate number of pauper patients in the metropolitan district.

"The effect of the death-rate in diminishing or augmenting the number of patients in asylums may be best shown by noting its operation in the two sexes. It is well known that the rate of mortality stands higher in the male than in the female wards; but the full effect of the greater or less fatality of the disease in lessening or increasing the number of the insane population, can only be fully estimated by observing the changes among the inmates of asylums during a long period of years.

"In illustration of this, we propose to review the movement of the population in the county and borough asylums, as shown in the tables now published. From these it appears that in the five years ending January 1st, 1859, a larger number of men than of women were admitted, and during the same period 700 more women than men were discharged; from which it might be presumed that a corresponding diminution would be the result. We find, however, that at the end of that period, there were 227 more women than men patients as compared with the relative number at its beginning. Thus, by reason of a lower rate of mortality among women, although a smaller number were admitted and a larger number discharged, yet in relation to the number of men there has been a considerable increase. In fact, as 700 more women than men were discharged, the lower death-rate among the women may be said to have augmented the number of this sex by nearly 1000 during a period of five years.

"In the general population of the country a somewhat analogous effect results from a like cause. A much larger number of boys than girls are born every year; and yet, owing to the greater rate of mortality among the former, the result is a considerable preponderance of women."

From the foregoing considerations, the Commissioners are inclined to believe, that the great accumulation of pauper patients, as shown by their own records and those of the Poor-Law Board, is mainly attributable:—

1st. To the more complete collection of annual returns, formerly very defective in this respect.

2nd. To the detection and registration of cases formerly left unnoticed.

3rd. To the removal of a larger proportion of patients from localities where they were exposed to causes of death, into asylums favouring the prolongation of life.

4th. To the effect of sanitary regulations in asylums, of improved diet, and of various means of sustaining the health and promoting the longevity of the entire body of inmates.

5th. To a like effect on those out of asylums, from the removal of large workhouses to more healthy sites, and from the medical visitation of such of the insane paupers as are neither in workhouses nor asylums.

The Commissioners then proceed thus:—

"Though it might, on the other hand, be fairly supposed that the increased proportion of cures in recent cases sent to asylums, caused by the improved modes of treatment now adopted, would have had the effect of diminishing the aggregate numbers resident, this latter cause of decrease in the comparatively few recent cases admitted, has apparently been more than counterbalanced by the prolongation of the lives of the many chronic cases brought under care.

"And as in certain localities unprovided with any recognised means of sheltering their insane paupers, we have shown that their seeming proportion to the population is small, so if we look to other countries we shall find that in proportion to the amount of the provision made by the State, will be the apparent ratio of the insane to the population.

"Considerations such as these furnish abundant reason for discrediting the statements which foreign authors have founded on our returns, to the effect that the inhabitants of this country are more liable to insanity than those of any other civilized state.

"Having thus indicated what we believe to be the causes of the great apparent accumulation of pauper patients, we come to deal practically with the fact that such causes being still in operation, we can only calculate on a further progressive increase; and we accordingly draw attention to the subject, in the hope that committees of visitors will see the necessity of a timely increase of accommodation. Whether some means might not also be adopted for effecting a legal transfer of some of the harmless and chronic pauper cases to the care of relatives, with provision at the same time for securing the payment of an adequate sum for their care, maintenance, and medical visitation, is, we think, matter for consideration deserving the attention of the Legislature.

"At a time when so large an amount of public accommodation exists, it may seem unreasonable to urge the necessity of making further provision; nevertheless, the returns before us show the importance of such a step, for the admissions into county and borough asylums during the latter half of the ten years under review, appear to have been in excess of the former half, to the extent of nearly 3000. During the five years ending 1853, 20,544 cases were admitted; and during the five years ending 1858, the numbers were 23,256; showing a steady increase in the influx of patients into our public asylums."

The Commissioners next observe that, even during the past year only, the number of patients in county and borough asylums has advanced to the extent of no less than 1151; and, finally, they conclude this most important portion of their Report in the following words:—

"It might be supposed that the admission of so many patients into asylums would be followed by a corresponding, or at all events a considerable, diminution of those placed or retained in workhouses, or receiving out-door relief. So far from this, however, being the case, the subjoined return shows that the insane poor, whether in workhouses or as single patients, have not diminished, but on the contrary have increased in number to a very considerable extent; a circumstance attributable in all probability, as we have endeavoured to point out, to the more complete registration of cases, and the greater attention which has been paid to their well-being of late years."

Assuming the probable correctness of the foregoing conclusions of the Commissioners in Lunacy, and that there is no sufficient evidence of an increased tendency to insanity among the population, the difficulties to be contended with in providing for our lunatics are in no degree abated. The actual condition of things in this respect is, indeed, just the same as if there were a real, progressive increase in the amount of lunacy in the kingdom. But the importance of ascertaining the true state of lunacy beyond the walls of our asylums and workhouses becomes still more apparent. We have often urged the need which exists for this knowledge, and we must perforce return to the subject again. Either our asylum accommodation has not been sufficiently extended to exhaust the substratum of chronic lunacy existing in the kingdom, or the increase of accommodation has taken place at so slow a rate, that it has never at any time overtaken the wants of the population. If the latter be the case, a large proportion of lunatics have been passing into a chronic state contemporaneously, and in about an equal degree, with the increase of accommodation.

Hitherto we have been content to deal with difficulties as they arose by meeting the immediate requirements of the moment and no more; and it is proposed to meet our present great difficulty, the crowding of asylums with chronic cases, and their consequent inefficiency as curative institutions, in the same fashion. Our asylums are, in fact, chiefly lunatic receptacles—asylums not in the sense of hospitals for cure, but hospitals for life-provision. The suggestion to provide other, and above all subsidiary (overflow) asylums, is no doubt necessary, but it is, after all, but a palliative recommendation. If, as the Commissioners assert, the chief sources of the increasing population of asylums rest in the measures which gave rise to their formation, and in the nature of the institutions themselves, it is evident that unless the accommodation provided by them be so widely extended as to afford at once a chance of exhausting the dead weight of chronic lunacy existing at large in the kingdom, and of thus bringing us face to face with the nascent lunacy, those sources will remain fully operative. At present, we simply extend our asylum accommodation proportionately to the evils (that is, evils in relation to the failure of asylums as means for checking and controlling the increase of the lunatic population) arising out of the system, and *not the good.*

That counties, and boroughs, and cities should hesitate to commit themselves to an expenditure, which, as the Commissioners put it, may have an almost unlimited growth, and should, as a rule, confine themselves to such provision for the insane as is absolutely necessary, is very natural. For our present system of attempting to deal with pauper lunacy is very similar to that of an individual who might seek to dry up or check the course of a stream by lading the waters, and storing it in reservoirs, at the mouth. We have great reservoirs of lunacy, and solicit the stream of lunatics to flow into them. We find after twenty years that our reservoirs, new and old, are full to overflowing, but that there is no sign of abatement in the flow of the stream of lunacy. We, however, persist in crying out for more reservoirs, and display a most praiseworthy energy in seeing to the integrity of those which exist, and to the most fitting state of the contents thereof. But let us note briefly the rapidity of motion of the stream and the capacity of our reservoirs for its reception.

The rate of increase in pauper lunacy (including idiots) during the ten years 1847-57 was 4 *per cent. per annum*, that is to say, nearly four times the rate of increase of population, and of such magnitude that, if it persists, the number of pauper lunatics in the kingdom would be *doubled in twenty-five years.* The rate of increase of pauper lunatics in county and borough asylums in 1857 was such that, if it were maintained, the whole of the then present and prospective asylum accommodation would be filled in five years.* We now learn from the Commissioners in Lunacy, that as the causes of this great increase in our lunatic population may be said to be inherent in our system of dealing with pauper lunatics as at present carried out, we may anticipate (as the Commissioners themselves point out) a persistence of this increase; and we are instructed to meet it by a continuation of

* See *Journal of Psychological Medicine* for full details on this question, vol. xii, p. 340, *et seq.*

precisely the same system of management under which the increase is occurring, and to which it is partly attributed—a system which has for results, that for every individual lunatic cured, two, it may said, at a rough guess, become life-pensioners on the bounty of the public!

That this is a necessary consequence if the difficulties besetting the question be completely grappled with, we do not for a moment believe. But how is it possible to grapple with them without knowing all the data necessary for their solution? It cannot surely be that we are to go on from year to year expending immense sums in carrying out a system of provision for our lunatics, a chief result of which is to augment the lunatic population of the country. Is it to be believed that this is a necessary result of an effective scheme of dealing with pauper lunacy? That it is the result of the present scheme we now know on the authority of the Commissioners of Lunacy themselves; and we frankly confess that we can see no help for it so long as we remain in ignorance of the actual state of the lunatic population at large in the kingdom. It is from this population that the crowds of chronic cases come in the first instance which fill our asylums, and interfere so seriously with their utility as curative institutions, hence bringing about a state of things which fosters chronic lunacy among lunatics at large. Until, therefore, we know the actual extent of the floating lunatic population, it is hopeless to imagine that any scheme can be developed which affords a reasonable hope of overcoming the difficulties which surround our present methods of dealing with pauper lunatics, or which would afford fair promise of ultimately holding in check the increase of pauper lunacy.

It is just to suppose that the diminution in the amount of lunacy among the wealthier classes of the population, is due in no small degree to the fact, that the provision made for the lunatics among these classes by private enterprise has been commensurate with the requirements of the case. It is just also to suppose that a similar result would follow from a like provision for pauper lunatics. But as private enterprise is sure to be developed proportionately to the field opened out for its exertion, it follows that it has an inherent power of adapting itself to the requirements of a people which public enterprise (so to speak) does not possess. For while the private individual limits his enterprise solely by the amount of present profit, as calculated by personal gain derived from it; a public body is of necessity restrained in its action by the vaguer, less defined, and often variable character of notions of public gain, and by its being responsible not to itself merely, but to the behests of those whom it represents. Now, it is not to be expected that it either can, or will, or should act effectually without being fully possessed of all the facts upon which its action is required; or if it does act in the absence of such facts, that action must of necessity be imperfect, which is the case with the doings of our public bodies in the case of pauper lunacy.

We presume that the facts recorded by the Commissioners of Lunacy respecting private patients will somewhat modify the opinions of those who doubt the utility of private asylums. It is dangerous to the public weal at all times to tamper with legitimate private enterprise; and private lunatic asylums are no exceptions to the rule.

It is unavailing to regret now that the details on the increase of patients in asylums, contained in the present Report of the Commissioners in Lunacy, were not laid before the Select Committee. It is difficult to conceive that the Committee could, had this been done, have omitted to observe the extreme importance, if it were in an economical point of view only, of ascertaining the true status of lunacy among our population. It is difficult to conceive also that the Committee would not have reported upon the necessity of an inquiry directed to this end, and the facility offered by the then approaching Census of carrying such an inquiry into effect. Not that the Census would have given all the information required, but it would have given, in the best and most economical manner possible, the data needed, as a basis for more elaborate local inquiries. Ultimately this inquiry must be conducted at the time of the Census, and by the same machinery, or by a special Government Commission, and it is not easy to see how this could possibly do the work required; for its investigations must, in the first place, be as widely extended as those of the Census. Sooner or later, however, the vast economical considerations, increasing year after year in weight and moment, must force this question upon the attention of the public and the Government.

The statistical tables appended to the present Report of the Commissioners in continuation of those contained in the eighth Report, show (1), the number of patients admitted into and discharged (by death or otherwise) from the asylums of England and Wales during a period of five years ending 31st December, 1858; and (2) the ages of all patients on the books on the 1st of January, 1854, and on the 31st of December, 1858, respectively; also the ages (in quinquennial periods) on admission, at death of all patients who were admitted or who died during the five years ending 31st December, 1858. In these tables the different classes of asylums are distinguished. A summary is also added relative to patients admitted during the year 1857, showing the numbers discharged, and distinguishing those who were recovered, the number of deaths and the number of patients remaining under treatment in February, 1860, the whole being grouped according to the duration of the disease on admission. From this summary we obtain the following important results confirming the great moment of early treatment in cases of lunacy:—

"Of 100 patients admitted in 1857, the duration of whose attacks at the time of admission did not exceed 1 month, 5·09 (or 509 in 1000 were discharged recovered, 9·7 (or 97 in 1000) were discharged relieved, 5·8 were discharged not improved, 17·8 (or 178 in 1000) died during the three years, and 15·3 were remaining at the end of the 3 years. Of 100 patients, the duration of whose attacks was 1 month and under 3, 45·4 were discharged recovered, 19·5 died, and 16·5 were remaining at the end of 1859. At 3 and under 6 months the percentage of recoveries was 35·2, of deaths 23·0, and of remaining 22·5. At 1 and under 3 years 15·8 per cent. recovered, 26·1 died, and 34·3 were left under treatment. Of 100 patients admitted, the period of duration of whose attacks was under 1 year, 44 recovered, 20 died, and 18 were remaining. At 1 and under 6 years, 14·5 per cent. recovered, 24·9 died, and 37·4 per cent. were remaining. At 6 years and upwards, 5·0 per cent. recovered, 17·3 per cent. died, and 63·3 per cent. were remaining under treatment at the end of the three years.

"From these results it would appear that the chances of recovery are much greater when the patients are placed under treatment in an early stage of the attack than they are when the disease has been allowed to remain unchecked for some time. The recoveries decrease with the increase of the period of duration of the attack at the time of coming under treatment, while the deaths increase as the period of duration of the attack rises to 1 and under 3 years, after which they decrease slightly in the next two periods.

"The proportion per cent. of patients remaining under treatment at the end of the three years increases progressively from 15 per cent. at the period of duration of under one month, to 63 per cent. at the period of duration of 6 years and upwards."

We need not dwell at any length on the remaining portions of the Report. It is chiefly occupied by detailed statements of the condition of several county and borough asylums, hospitals, and licensed houses, noting the advances which have been made or shortcomings recorded in 1860. Several cases are also noted in further illustration of the opinions expressed by the Commissioners in the supplement to their twelfth Report, on the many evils arising from the present indiscriminate use of workhouses as receptacles for persons of unsound mind. Other cases of neglect, improper, or ill treatment, are reported at length. The subject of single patients is further pursued, and several examples of gross neglect brought to light. On the probationary treatment of patients, the Commissioners remark:—

"Some idea may be formed of the extent to which we avail ourselves of the power vested in us by the 86th section [permitting the absence of patients from asylums on trial], when we state that about one-fourth of the whole number of private patients resident in the houses licensed by us, have been sent out on trial, or leave of absence, during the past year."

Certain changes have taken place in the Lunacy Board since the previous Report of the Commissioners. Lord Lyveden has resigned his seat at the Board. Mr. Procter (after 28 years' service on the present and previous Boards) has also resigned his seat, but has been appointed an unpaid commissioner. Mr. Forster, who has occupied the post of secretary for upwards of five years, has succeeded Mr. Procter as a legal commissioner; and the Hon. W. C. Spring Rice has been appointed Secretary.

One new asylum was opened in 1860, the asylum for the united counties of Bedfordshire, Hertfordshire, and Huntingdonshire, and when visited by the Commissioners on the 16th April, 1860, it contained 323 patients. Several asylums have been enlarged.

We add the tabulated summary of admissions to public and private asylums and hospitals in 1857, and the results in the cases so admitted during the three years 1857-58-59; also the summary, showing the state of the lunatic population of the different asylums throughout England and Wales in 1860; and the general summary for the five years ending 31st December, 1858.

---

TABLE showing the NUMBER of PATIENTS ADMITTED into the LUNATIC ASYLUMS, HOSPITALS, and LICENSED HOUSES in *England* and *Wales* during the year 1857; also the proportion per Cent. of the Patients so admitted in 1857 who were Discharged during the Three years 1857 to 1859, and also those who were Remaining at the end of 1859.

| Duration of Existing Attack at the Time of Admission. | Total Number of Patients Admitted during the year 1857. | Proportion per Cent. on Patients admitted in 1857. | | | | | | |
|---|---|---|---|---|---|---|---|---|
| | | DISCHARGED. during the Three Years 1857 to 1859 (inclusive). | | | | | | Patients Remaining at the end of 1859. |
| | | Recovered. | Relieved. | Not Improved. | Died. | Not stated. | Total Discharged. | |
| TOTAL . . . . . | 7,708 | 35·6 | 9·9 | 8·2 | 19·6 | ·7 | 74·0 | 26·0 |
| Under 1 month . . | 2,209 | 50·9 | 9·7 | 5·8 | 17·8 | ·5 | 84·7 | 15·3 |
| 1 and under 3 months | 1,505 | 45·4 | 11·3 | 6·5 | 19·5 | ·8 | 83·5 | 16·5 |
| 3 ,, 6 ,, . | 825 | 35·2 | 9·6 | 9·0 | 23·0 | ·7 | 77·5 | 22·5 |
| 6 ,, 12 ,, . | 575 | 28·9 | 8·7 | 12·7 | 24·9 | ·5 | 75·7 | 24·3 |
| 1 ,, 3 years . | 633 | 15·8 | 13·1 | 8·8 | 26·1 | 1·9 | 65·7 | 34·3 |
| 3 ,, 6 ,, . | 234 | 11·1 | 12·4 | 9·0 | 21·8 | ... | 54·3 | 45·7 |
| 6 years and upwards . | 444 | 5·0 | 6·1 | 7·9 | 17·3 | ·4 | 36·7 | 63·3 |
| Not stated . . . . | 1,283 | 25·6 | 8·6 | 11·8 | 15·2 | ·9 | 62·1 | 37·9 |
| Under 1 year . . . | 5,114 | 44·3 | 10·0 | 7·3 | 19·9 | ·6 | 82·1 | 17·9 |
| 1 and under 6 years . | 867 | 14·5 | 12·9 | 8·9 | 24·9 | 1·4 | 62·6 | 37·4 |
| 6 years and upwards . | 444 | 5·0 | 6·1 | 7·9 | 17·3 | ·4 | 36·7 | 63·3 |

| | Number of Patients, 1st January, 1860. | | | | | | | Admissions during the Year 1860. | | | Discharges during the Year 1860. | | | | | | Deaths during the Year 1860. | | | | | | | | |
|---|---|---|---|---|---|---|---|---|---|---|---|---|---|---|---|---|---|---|---|---|---|---|---|---|---|
| | Private. | | | Pauper. | | | Total Lunatics. | | | | Total Number. | | | Number Recovered. | | | Total Number. | | | From Suicide: Act committed in Asylum. | | | From Suicide: Act committed before admission. | | |
| | M. | F. | Total. | M. | F. | Total. | | M. | F. | Total. | M. | F. | Total. | M. | F. | Total. | M. | F. | Total. | M. | F. | Tot. | M | F | Tot. |
| County and Borough Asylums | 121 | 106 | 227 | 7,830 | 9,379 | 17,209 | 17,436 | 3,169 | 3,457 | 6,626 | 1,461 | 1,819 | 3,280 | 894 | 1,138 | 2,032 | 1,281 | 914 | 2,195 | 7 | 4 | 11 | ... | 1 | 1 |
| Hospitals | 1,000 | 752 | 1,752 | 120 | 113 | 233 | 1,985 | 485 | 444 | 929 | 309 | 312 | 621 | 163 | 167 | 330 | 96 | 53 | 149 | — | 1 | 1 | ... | ... | ... |
| Metropol. Licensed Houses | 703 | 639 | 1,342 | 194 | 408 | 602 | 1,944 | 379 | 433 | 812 | 258 | 331 | 589 | 111 | 139 | 250 | 128 | 86 | 214 | 2 | 3 | 5 | ... | ... | ... |
| Provincial Licensed Houses | 874 | 732 | 1,606 | 377 | 373 | 750 | 2,356 | 472 | 401 | 873 | 408 | 480 | 880 | 135 | 158 | 293 | 110 | 81 | 191 | 3 | 1 | 4 | ... | ... | |
| Totals | 2,698 | 2,229 | 4,927 | 8,521 | 10,273 | 18,794 | 23,721 | 4,505 | 4,735 | 9,240 | 2,436 | 2,942 | 5,378 | 1,303 | 1,602 | 2,905 | 1,615 | 1,134 | 2,749 | 12 | 9 | 21 | ... | 1 | 1 |

| | Patients remaining 1st January, 1861. | | | | | | | | | | | | | | | | | | | |
|---|---|---|---|---|---|---|---|---|---|---|---|---|---|---|---|---|---|---|---|---|
| | Private. | | | Pauper. | | | Total Lunatics. | Number deemed Curable. | | | Found Lunatic by Inquisition. | | | Criminals. | | | Chargeable to Counties or Boroughs. | | |
| | M. | F. | Total. | M. | F. | Total. | | M. | F. | Total. | M. | F. | Total | M. | F. | Total | M. | F. | Total. |
| County and Borough Asylums | 108 | 104 | 212 | 8,270 | 10,105 | 18,375 | 18,587 | 761 | 1,104 | 1,865 | 13 | 4 | 17 | 268 | 113 | 381 | 756 | 924 | 1,680 |
| Hospitals | 1,073 | 813 | 1,886 | 127 | 131 | 258 | 2,144 | 149 | 209 | 358 | 20 | 16 | 36 | 120 | 22 | 142 | ... | ... | ... |
| Metropolitan Licensed Houses | 727 | 653 | 1,380 | 163 | 410 | 573 | 1,953 | 109 | 159 | 268 | 67 | 61 | 128 | 20 | 3 | 23 | 29 | 44 | 73 |
| Provincial Licensed Houses | 921 | 717 | 1,638 | 284 | 228 | 512 | 2,150 | 157 | 158 | 315 | 78 | 42 | 120 | 194 | 48 | 242 | 45 | 13 | 58 |
| Totals | 2,829 | 2,287 | 5,116 | 8,844 | 10,874 | 19,718 | 24,834 | 1,176 | 1,630 | 2,806 | 178 | 123 | 301 | 602 | 186 | 788 | 830 | 981 | 1,811 |

Included in Total Lunatics.

*TABLE showing the Mean Population, Number of Patients treated, and the Deaths, in County and Borough Asylums, Hospitals, and Licensed Houses; also the Average Annual Rate of Mortality per Cent., and the Mean Term of Treatment of Patients, during the Five Years ending 31st December, 1858.*

| AGES. | Mean Population (=Mean of the Numbers on the Books on 1st January, 1854 and 31st December, 1858). | | | Estimated Number of Patients treated during the Five Years 1854-58. | | | Deaths of Patients during the Five Years 1854-58. | | | Average Annual Rate of Mortality per Cent. during the Five Years 1854-58. | | | Mean Term of Treatment, expressed in Years, of each Patient treated, during the Five Years 1854-58. | | |
|---|---|---|---|---|---|---|---|---|---|---|---|---|---|---|---|
| | Males. | Females. | TOTAL. | Males. | Females. | TOTAL. | Males. | Females | TOTAL. | Males. | Females | TOTAL. | Males. | Females | TOTAL. |
| | | | | | | | | | | | | | *Years.* | *Years.* | *Years.* |
| All ages - - | 9,460 | 10,765 | 20,225 | 17,678 | 17,974 | 35,652 | 6,269 | 4,821 | 11,090 | 13·25 | 8·96 | 10·97 | 2·68 | 3·00 | 2·84 |
| Under 15 years - | 110 | 77·5 | 187·5 | 266 | 172·5 | 438·5 | 38 | 22 | 60 | 6·91 | 5·68 | 6·40 | 2·07 | 2·25 | 2·14 |
| 15 and under 20 | 243·5 | 216 | 459·5 | 880·5 | 921 | 1,801·5 | 101 | 71 | 172 | 8·30 | 7·57 | 7·49 | 1·38 | 1·17 | 1·28 |
| 20 " 25 | 576·5 | 515 | 1,091·5 | 1,604·5 | 1,791 | 3,395·5 | 239 | 217 | 456 | 8·30 | 8·43 | 8·36 | 1·80 | 1·44 | 1·61 |
| 25 " 30 | 884 | 833·5 | 1,717·5 | 1,706 | 2,032·5 | 3,828·5 | 357 | 273 | 630 | 8·08 | 6·55 | 7·34 | 2·46 | 2·05 | 2·24 |
| 30 " 35 | 1,115 | 1,071 | 2,186 | 2,201 | 2,209 | 4,410 | 680 | 410 | 1,090 | 12·20 | 7·66 | 9·97 | 2·53 | 2·42 | 2·48 |
| 35 " 40 | 1,162·5 | 1,173 | 2,335·5 | 2,186·5 | 2,017 | 4,203·5 | 803 | 482 | 1,285 | 13·82 | 8·22 | 11·00 | 2·66 | 2·91 | 2·78 |
| 40 " 45 | 1,113·5 | 1,279·5 | 2,393 | 2,043·5 | 1,996·5 | 4,040 | 830 | 458 | 1,288 | 14·91 | 7·16 | 10·76 | 2·72 | 3·20 | 2·96 |
| 45 " 50 | 928·5 | 1,187 | 2,115·5 | 1,617·5 | 1,613 | 3,230·5 | 669 | 444 | 1,113 | 14·41 | 7·48 | 10·52 | 2·87 | 3·68 | 3·27 |
| 50 " 55 | 802·5 | 1,099·5 | 1,902 | 1,427·5 | 1,509·5 | 2,937 | 581 | 398 | 979 | 14·48 | 7·24 | 10·29 | 2·81 | 3·64 | 3·24 |
| 55 " 60 | 655·5 | 902·5 | 1,558 | 974·5 | 957·5 | 1,932 | 468 | 404 | 872 | 14·28 | 8·95 | 11·19 | 3·36 | 4·71 | 4·03 |
| 60 " 65 | 492 | 677·5 | 1,169·5 | 883 | 942·5 | 1,825·5 | 433 | 414 | 847 | 17·60 | 12·22 | 14·48 | 2·79 | 3·59 | 3·20 |
| 65 " 70 | 323 | 478·5 | 801·5 | 564 | 576·5 | 1,140·5 | 362 | 412 | 774 | 22·41 | 17·22 | 19·31 | 2·86 | 4·15 | 3·51 |
| 70 and upwards - | 330·5 | 524 | 854·5 | 583·5 | 665 | 1,248·5 | 515 | 675 | 1,190 | 31·16 | 25·76 | 27·86 | 2·83 | 3·94 | 3·42 |
| Not stated - - | 723 | 730·5 | 1,453·5 | 650 | 570·3 | 1,220·5 | 195 | 141 | 334 | 5·34 | 3·86 | 4·60 | 5·56 | 6·40 | 5·96 |

*Explanation of the foregoing Table.*

The mean population is obtained by taking the mean of the number of patients on the books at the beginning and end of the five years, thus:—

| | |
|---|---|
| Patients on the books on 1st January, 1854 . | 18,195 |
| ,, ,, ,, 31st December, 1858 . | 22,255 |
| | 2)40,450 |
| Mean Population . | 20,225 |

The estimated number of patients treated in the five years is obtained by taking the mean of the numbers admitted and discharged during that period, thus:—

| | |
|---|---|
| Patients on the books on 1st January, 1854 . | 18,195 |
| Patients admitted in the five years ending 31st December, 1858 . . . . . . . . . . . | 37,682 |
| | 55,877 |
| Patients remaining on the books on 31st December, 1858 . . . . . . . . . . . | 22,255 |
| Patients discharged in the five years . . . . | 33,622 |
| Patients admitted in the five years . . . . . | 37,682 |
| | 2)71,304 |
| Patients treated in the five years . . . . . | 35,652 |

The average annual rate of mortality is obtained by dividing the deaths in the five years by five times the mean population.

The mean term of treatment of each patient treated during the five years is obtained thus:—

$$\frac{\text{Mean population} \times 5}{\text{Numbers treated in the five years}} = \frac{101,125}{35,652} = 2{\cdot}84 \text{ years.}$$

